# High BMI and the risk for incident type 1 *Diabetes Mellitus*: a systematic review and meta-analysis of aggregated cohort studies

**DOI:** 10.1186/s12933-023-02007-y

**Published:** 2023-11-02

**Authors:** Maya Nitecki, Hertzel C. Gerstein, Yulia Balmakov, Eyal Tsur, Vladislav Babushkin, Tomer Michaeli, Arnon Afek, Orit Pinhas-Hamiel, Tali Cukierman-Yaffe, Gilad Twig

**Affiliations:** 1 Israel Defense Forces, Medical Corps, Ramat Gan, Israel; 2 Department of Military Medicine, Faculty of Medicine, Hebrew University of Jerusalem, Jerusalem, Israel; 3 Department of Preventive Medicine, School of Public Health, Sackler Faculty of Medicine, Tel Aviv University, Tel Aviv, Israel; 4 Department of Medicine, McMaster University, Hamilton, ON Canada; 5 Central Management, Sheba Medical Center, Ramat Gan, Israel; 6 Sackler Faculty of Medicine, Tel Aviv University, Tel Aviv, Israel; 7 Pediatric Endocrine and Diabetes Unit, Edmond and Lily Safra Children’s Hospital, Sheba Medical Center, Ramat Gan, Israel; 8 Institute of Endocrinology Diabetes and Metabolism, Sheba Medical Center, Ramat Gan, Israel; 9 The Gertner Institute for Epidemiology & Health Policy Research, Sheba Medical Center, Ramat Gan, Israel

**Keywords:** Type 1 diabetes, Obesity, Meta-analysis, Cohort studies

## Abstract

**Background:**

There is uncertainty regarding the role of obesity in type 1 diabetes development. The aim of this systematic review and meta-analysis was to collect and synthesize evidence regarding BMI and the risk of developing type 1 diabetes.

**Methods:**

A systematic review and meta-analysis were conducted to assess the association between BMI and incident type 1 diabetes. Databases were searched up to June 2022. Cohort studies were included reporting the association between overweight and/or obesity, as measured by BMI after age 2 years, with incident type 1 diabetes. Independent reviewers extracted data and assessed study quality. Risk estimates were pooled using a random-effects model.

**Results:**

Ten cohort studies met the inclusion criteria. The seven studies that classified BMI into categories were of high quality and involved 1,690,660 individuals and 1979 incident type 1 diabetes cases. The pooled risk ratio (RR) for type 1 diabetes was 1.35 (95% CI 0.93–1.97) among people with overweight (3 studies); 2.17 (95% CI 1.75–2.69) among people with obesity (5 studies); and 1·87 (95% CI 1.52–2.29) among people with overweight/obesity (two studies merged the categories). These point estimates persisted in sensitivity analyses that addressed the duration of follow-up, variability in baseline risk for incident type 1 diabetes, and potential misclassifications related to exposure or outcome definitions. People with overweight/obesity had a 2.55 (95% CI 1.11–5.86) greater risk for incident type 1 diabetes with positive islet autoantibodies.

**Conclusion:**

This systematic review and meta-analysis of high-quality observational cohort studies indicated an association between high BMI and the risk of type 1 diabetes, in a graded manner.

**Supplementary Information:**

The online version contains supplementary material available at 10.1186/s12933-023-02007-y.

## Background

Type 1 diabetes is a chronic autoimmune disease characterized by insulin deficiency and resultant hyperglycemia [1]. The peak incidence occurs in the age range of 10–14 years; 25% develop it during adulthood [2]. Twin studies have supported a genetic heritable basis for disease development, showing 30–70% identical twin concordance [3]. Yet, in recent decades, several observations have also suggested that modifiable environmental factors such as obesity may have a role. These include ecological studies [4, 5] that showed a growing incidence of type 1 diabetes in parallel with the global increase in obesity rates, and a meta-analysis [6] that reported a positive association between obesity and type 1 diabetes. However, the inclusion of case-control studies within the meta-analysis, misclassification of diabetes type and the absence of distinction between childhood obesity and other risk factors such as birth weight, continue to drive uncertainty regarding the robustness of any obesity-type 1 diabetes relationship. We therefore conducted a systematic review of cohort studies, to synthesize evidence regarding the relationship between BMI after infancy and incident type 1 diabetes.

## Methods

### Literature search

For this systematic review and meta-analysis, we searched the published literature using the Ovid MEDLINE & EMBASE databases (from inception to June 13, 2022). The search structures, Medical Subject Headings (MeSH), and keywords were tailored to each database by a medical research librarian with experience in systematic reviews. The terms “type 1”, “early onset”, “juvenile”, and “insulin dependent”, were used to identify type 1 diabetes. The complete MEDLINE and EMBASE search strings are listed in Additional file 1: Table S1. The search was not limited by language or publication date. Abstracts of the articles identified by the search were read and evaluated on the basis of the inclusion criteria. Reference lists of the included articles were searched manually. A list of the included articles was sent to experts in the field for their review. Our findings are reported in accordance with the Preferred Reporting Items for Systematic Reviews and Meta-Analyses (PRISMA) reporting guidelines [7]. The study protocol is registered in PROSPERO (CRD42023417321).

### Study selection

After the initial search, five reviewers (M.N, Y.B, T.M, E.T, and V.B) independently screened the titles and abstracts of the articles to identify potentially relevant studies. Two votes per reference were required in each screening stage, and three in the presence of conflicts. The full text was reviewed of all the studies whose titles and abstracts were potentially relevant. The same reviewers screened the full text-articles to apply inclusion and exclusion criteria. Disagreements were resolved by consensus or by seeking the opinion of M.N.

### Eligibility criteria

Observational cohort studies were included in the meta-analysis if they met all the following inclusion criteria. (i) The exposure was overweight, obesity, or both, as measured by BMI at a single time-point. For studies that reported repeated BMI measurements, we used the baseline BMI in the analysis. (ii) The studies included BMI data after the age of 2 years. This cutoff was chosen because anthropometric measures before this age are not reported as BMI. Also, the incidence of type 1 diabetes before age 2 years is low, and we intended to focus on potential modifiable lifespan factors rather than on those related to the intrauterine environment. While possibly inferior to other adiposity indexes, BMI is well-studied and was recommended by the US Preventive Services Task Force as the screening measure of choice for childhood and adolescent obesity [8]. Further, BMI is the measure preferred by the American Diabetes Association (ADA) for weight surveillance among individuals with pre-diabetes or diabetes [9]. (iii) Cohort studies that followed individuals for the development of type 1 diabetes, as defined by each study.

Studies were excluded if they met any of eight exclusion criteria. (i) A case–control study or another non-cohort study design. (ii) Measurement of the exposure by a method other than BMI. (iii) Definition of the exposure as a change in BMI or its velocity between several time points, with no available baseline BMI data or effect estimate for baseline BMI. (iv) A mean or median follow up of less than 12 months. (v) The inclusion at baseline of individuals with the diagnosis of diabetes mellitus. (vi) An outcome of type 2 diabetes or unspecified diabetes mellitus. (vii) Duplication of the study population in another study that was included in the meta-analysis. When studies of duplicate populations were identified, we selected the study that was more inclusive of the original study population but collected relevant data from both.

### Quality assessment

The quality of each study was assessed using the Newcastle–Ottawa Scale (NOS) for evaluating the quality of nonrandomized studies [10]. The scale evaluates study bias and assigns points in the following domains: selection of participants, measures of exposure and outcome variables, and control of confounders. The scale yields a quantitative summary score and a qualitative categorization of quality (poor, fair, or good) based on the number of points in the three domains. Each study was assessed independently by two reviewers during the extraction phase, disagreements were resolved by M.N. As accepted in the literature[11, 12], study quality was considered high if the NOS score was at least 7 points, of a possible 9, and categorized as good. The latter required 3 or 4 points in the selection domain, 2 or 3 points in the exposure and outcome domain, and 1 or 2 points in the comparability domain. Otherwise, study quality was considered low.

### Data collection process

Two reviewers independently extracted data from each included study. These data included information regarding the study design, the study population, characteristics of the exposure and outcome measures, and measures of the association. Data of the study population included the baseline risk (high/average) for type 1 diabetes, the geographic region, demographic characteristics, the number of participants with overweight and obesity, mean or median BMI values, and the mean or median follow up period. The exposure and outcome characteristics included BMI classification (WHO, CDC, z-score, or other) and the documentation method (self-reported/measured), as well as the type 1 diabetes definition and documentation method employed by each study. When available, the number of cases in each BMI category was extracted. Data regarding the association included the adjusted hazard ratio (HR) or risk ratio (RR), with a 95% confidence interval (CI) and the variables used for adjustment. These data were validated by M.N. When data were unavailable in the formal publication, efforts were made to contact the corresponding author and obtain the missing data.

### Statistical analysis

Summary statistics from the included studies were used because the raw data were not available. Accordingly, the HRs or RRs, and the 95% CIs from the published papers or supplementary material were used. Estimates from each study were combined by using inverse variance-weighted averages of logarithmic RRs in a random-effects analysis [13]. The random-effect model was chosen to allow for between-study variability, which may arise from the length of follow-up, exposure categorization, outcome measures, and model adjustment. This model was used to investigate whether increased BMI was associated with incident type 1 diabetes. Pooled RRs and 95% CIs were estimated to compare the risk of developing type 1 diabetes in individuals with overweight or obesity, to the risk of the normal reference group, based on the reported measures of association. When obesity and overweight were considered together, the combined category was referred to as overweight/obesity. The definitions of overweight and obesity that were used for each study included in the analysis are mentioned in the figure legends. Additionally, we analyzed the increase in the risk of type 1 diabetes per unit increment in BMI, based on studies that used BMI as a continuous variable. Heterogeneity was assessed among the included studies using the I^2^ statistic. I^2^ values of 25%, 50%, and 75% were considered low, moderate, and high heterogeneity, respectively [14]. Funnel plots were used to examine and visualize publication bias.

Several sensitivity analyses were performed. (i) To investigate whether any of the studies had a disproportionate influence on the results of the meta-analysis, the data were pooled after serially excluding each study included in the main analysis. (ii) To assess whether the results were sensitive to the choice of the meta-analysis model, a fixed-effects meta-analysis was performed separately. (iii) We assured that study selection in the case of duplicates did not bias the results.

We conducted several sub-group analyses, to explore the heterogeneity between the studies. We separately pooled studies that included populations at average risk for developing type 1 diabetes (i.e., excluding studies with high-risk populations: multiple autoantibody positivity, 1^st^ degree relatives with type 1 diabetes, or high-risk HLA genotypes) and studies that required the presence of autoantibodies for type 1 diabetes case ascertainment. Lastly, we divided the studies by age at enrollment (up to 11 years old vs. older) and by follow-up duration (up to 12 years vs. longer). The threshold of 12 years was chosen because it represents the mean duration of central tendency measures across all the included studies.

All analyses were performed with Review Manager (RevMan) [Computer program] Version 5.4. The Cochrane Collaboration, 2020. All the reported statistical tests were two-sided, with a significance level of 0.05.

## Results

**Fig. 1:**
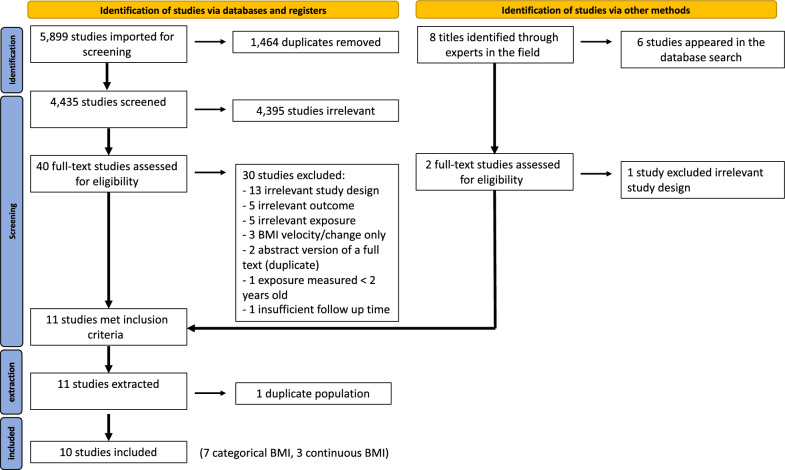
PRISMA flow diagram. PRISMA indicates Preferred Reporting Items for Systematic Reviews and Meta-analyses

The initial search identified 5899 references. After duplicates were removed, we abstracted 4435 unique references. Of these, 4395 were excluded based on the title and abstract, leaving 40 studies for full-text assessment. Overall, 30 studies were excluded after full-text review. Thirteen studies were excluded due to a non-cohort design, 5 due to irrelevant exposure, and 5 due to irrelevant outcome measures. Three studies reported a change in BMI or its velocity, without analysis of a single time-point BMI. Two references were abstracts of an included full text (duplicate), one had an insufficient median follow-up time, and one reported exposure data under the age of 2 years (Additional file 1: Table S2). In addition to the 10 studies that remained after the full-text assessment, eight studies were identified by approaching experts in the field. Of these, six were already identified and excluded as part of the process mentioned above, and the remaining two were evaluated in full text. One study was excluded due to an inappropriate design, and the other one met the inclusion criteria [15]. Consequently, 11 studies met our inclusion criteria and were extracted. Of these, 3 used BMI as a continuous variable only [16–18], and 8 applied BMI into categories [15, 19–25]. Duplicates were removed separately for studies in which BMI was continuous and for those in which BMI was applied into categories. After removal of duplicate populations (n = 1), 10 studies were included, 7 that evaluated BMI as a categorical variable and 3 that used continuous BMI data. The PRISMA flow chart (Fig. 1) shows the entire review process, from the original search to the final selection of studies.

The characteristics of all 10 studies that were included are summarized in Table 1. All the studies were of good quality according to the Newcastle–Ottawa Scale score (Table 2).

**Table 1:** Characteristics of the extracted studies (n = 10)

Authors	Geographic region	Sample size	Age (years)	Sex (female %)	Follow up (years)	Proportion with obesity	Type 1 diabetes definition	Number of cases (%)
Carlsson et al. [25]	Europe	38,800	40–59^b^	NR	11 years^a^	9.00%	All of the following: (1) Self-reported diabetes (2) Insulin treatment within 6 months of diagnosis (3) Positive anti-GAD or C-peptide < 150 p/mol	18 (0.05%)
Viner et al. [19]	Europe	11,261	10^c^	52.00%	20^c^	4.20%	Self-reported insulin-dependent diabetes before age 30 years	47 (0.42%)
Harpsøe et al. [24]	Europe	75,008	30.2 [27.4–33.3]^b^	100%	11.4 **[**10.2–12.5]^b^	8.1^d^%	First hospital contact (primary diagnoses on in- and outpatient contacts) with ICD-8–10 type 1 diabetes codes obtained from the Danish national patient register	144 (0.19%)
Tosur et al. [20]	North America, Australia, Europe	4,873	11.7–12.4 [1.06–51.3]^b^	52.20%	1.0 [0.4–2.9]^b^	15.2%^e^	ADA criteria, with the presence of antibodies	591 (12.13%)
Nucci et al. [21]	North America, Australia, Europe	2,149	Birth cohort, measurement at different time points	47.23%	10^c^	At age 2 years: 1.5% At age 10 years: 9.0%	WHO, some with the presence of antibodies	172 (8.00%)
Herzog et al. [23]	Europe	132,207	43.89 (14.39)^a^	48.25%	18.87 (5.87)^a^	34.26%^e,f^	All of the following: (1) National registry record (2) One of the following: - Age at diagnosis < 30 years - Insulin prescription	230 (0.17%)
Zucker et al. [15]	West Asia	1,426,362	17.3 (0.5)^b^	41.50%	11.1 (5.9)^a^	6.37%	All of the following: (1) National registry record (2) Actively using short-acting insulin (3) Treatment with short-acting insulin initiated within 1 year of diagnosis (4) No history of treatment with oral anti-glycemic drugs (Antibodies in a sensitivity analysis)	777 (0.05%)
Ferrannini et al. [16]	North America	328	11 [8]^b^	46.60%	3.2 [2.8]^b^	N/A^g^	ADA criteria, with the presence of antibodies	115 (35.06%)
Steck et al. [18]	North America	68	NR	52.90%	5.7^a^	N/A^g^	ADA criteria, with the presence of antibodies	25 (36.78%)
So et al. [17]	North America, Australia, Europe	3,856	13.27 (10.96)^a^	47.50%	TrialNet’s Pathway to Prevention Study cohort	N/A^g^	ADA criteria, with the presence of antibodies	896 (23.24%)

*NR* not reported, *ADA* American Diabetes Association. *WHO* World Health Organization, *NHW* non-Hispanic white, *NHB* non-Hispanic black. All BMI values are measured BMI unless indicated otherwise.

^a^Mean, SD

^b^Median [IQR / range]

^c^Birth cohort, included only those with full follow-up

^d^BMI was self-reported

^e^Proportion was reported as overweight or obesity

^f^BMI was collected from medical records

^g^Not applicable, as BMI was not applied into categories

**Table 2:** Association between BMI and type 1 diabetes and risk of bias across included studies (n = 10)

Study	Effect estimates overweight (95%CI)	Effect estimates obesity (95%CI)	Increment in risk for 1 unit of BMI	Adjusted to	NOS quality score
Carlsson et al. [25]	0.38 (0.10-1.36)^a^	1.16 (0.25–5.41)	0.92 (0.78–1.08)	Sex, age, smoking	7
Viner et al. [19]	-	3.1 (1.0–9.3)	1.8 (1.2–2.8)	Sex, maternal educational, birth weight, puberty stage, breastfeeding, social class	7
Harpsøe et al. [24]	1.42 (0.95–2.14)	2.67 (1.71–4.17)	1.07 (1.04–1.11)	Smoking, alcohol, parity, socio-occupational status	8
Tosur et al. [20]	1.37 (1.05–1.79)^b^	–	1.00 (0.997–1.004)	Sex, number of Abs (single vs. multiple), DPTRS, HLA-genotype	8
Nucci et al. [21]	1.26 (0.71–2.21)^b^	1.59 (0.50–5.08)	N/A^c^	Sex, birth weight z-score, birth length z-score, HLA risk, maternal type 1 diabetes, mode of delivery, breastfeeding duration (> \ < 6 months)	8
Herzog et al. [23]	1.88 (1.43–2.47)^b^	**–**	N/A^c^	Sex, age (timescale), calendar time, fasting status (overnight fasting vs. no fasting), socioeconomic status, country of birth	7
Zucker et al. [15]	1.54 (1.23–1.94)	2.05 (1.58–2.66)	1.25 (1.17–1.32)	Sex, age, birth year, education, cognitive score	8
Ferrannini et al. [16]	N/A^d^	N/A^d^	Females: 2.25 (1.31–3.79)^a^ Males: 1.27 (0.72–2.13)^a^	Age, ß-cell glucose sensitivity insulin sensitivity, projective 5-year risk of diabetes (high vs. moderate)	7
Steck et al. [18]	N/A^d^	N/A^d^	0.86 (0.72–1.02)	Unadjusted	7
So et al. [17]	N/A^d^	N/A^d^	1.2 (1.11–1.31)	Unadjusted	7

*NOS* Newcastle–Ottawa Scale, *Abs* antibodies; *DPTRS *Diabetes Prevention Trial-Type 1 Risk Score.

^a^RR (risk ratio).

^b^Hazard ratios were reported for overweight or obesity.

^c^Not applicable, as BMI was not modeled as a continuous variable.

^d^Not applicable, as BMI was not applied into categories.

### Categorical BMI data

The seven studies with categorical BMI data provide information on 1,690,660 men and women, and 1979 cases of incident type 1 diabetes (0.12%). The mean or median age at study entrance ranged between 2 and 59 years. Four studies included children and adolescents (< 20 years), while the other 3 included only adults (> 20 years). The sample sizes of the studies ranged from 2149 to 1,426,362. The reported mean or median duration of follow-up ranged between 1 and 20 years. Five studies collected measured BMI, one collected BMI from medical records, and one study used self-reported BMI. For the outcome definition, one study used self-reported insulin dependent diabetes mellitus. All the other studies (n = 6) used some form of record documentation for defining type 1 diabetes, based on physician diagnosis, listing in a national registry or International Classification of Disease (ICD) code of hospital contacts, or either lab results or insulin prescriptions (Table 1).

**Fig. 2:**
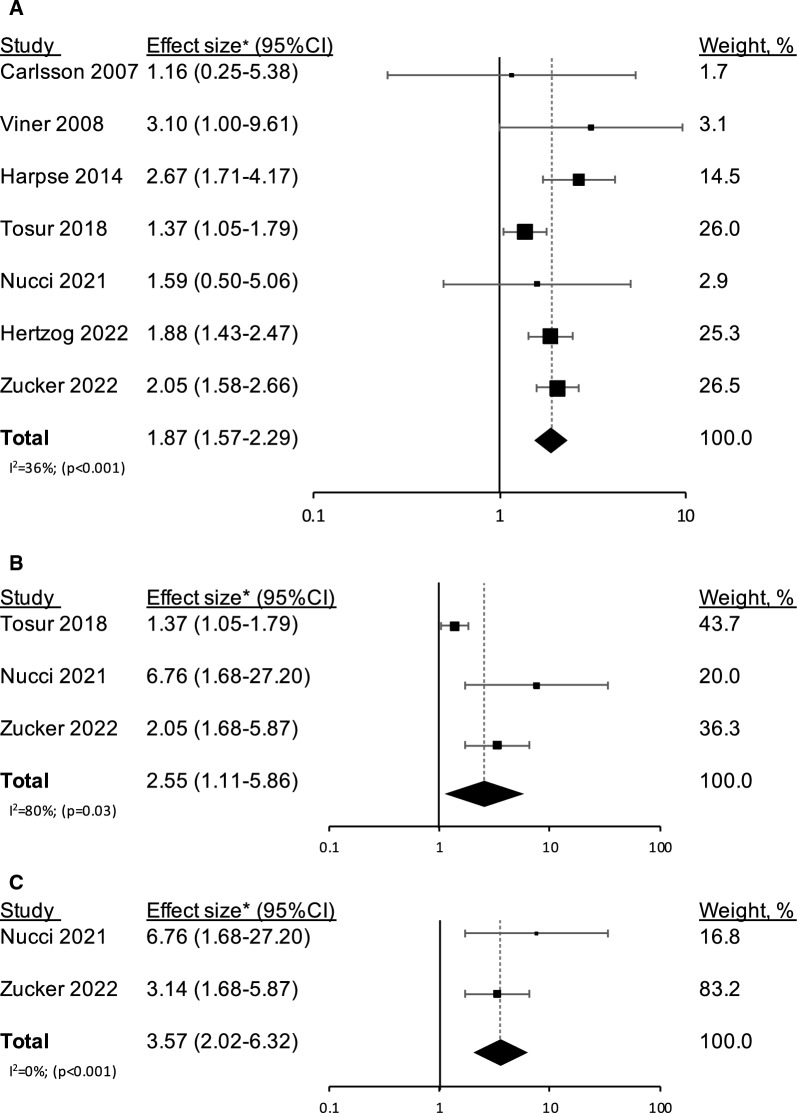
Confounder-adjusted effect sizes (95% CI) for incident type 1 diabetes among individuals with obesity or overweight/obesity compared to those without, are shown for individual studies and for pooled risk ratio results from the meta-analysis. The effect sizes are shown across all the studies (**a**) and across the studies that were limited to autoantibody-proven type 1 diabetes (**b**). Panel c shows the confounder-adjusted effect size (95% CI) for incident type 1 diabetes, limited to autoantibody-proven type 1 diabetes, in individuals with obesity. The sizes of the boxes correspond to the weights of the study in the meta-analysis. The diamond depicts the point estimate (95% CI). The vertical line is centered at the null. Overweight and obesity were defined as follows: Carlsson et al. and Harpsøe et al. [25, 24] —BMI ≥ 25 kg/m^2^ and BMI ≥ 30 kg/m^2^, Viner et al. and Nucci et al.[19, 21], —according to the International Obesity Task Force cut-off points by sex and age (corresponding BMI ≥ 25 kg/m^2^ and BMI ≥ 30 kg/m^2^ in adults), Zucker et al. [15], —85th ≤ BMI ≤ 94th percentiles and BMI ≥ 95th percentile. Tosur et al. [20] and Herzog et al. [23] merged overweight and obesity into a single category that was defined as BMI ≥ 25 kg/m^2^. Of note, Carlsson et al. [25] considered either anti-GAD presence or C-peptide levels < 150 pmol/L as confirmation of type 1 diabetes. Yet, as this was done in only 76% of self-reported type 1 diabetes cases, the study was excluded from the subgroup analysis in panels **b** and **c**. *All the studies reported hazard ratios, except Carlsson et al. [25] who reported risk ratios.

The RR for type 1 diabetes among the individuals with obesity or overweight/obesity was 1.87 (1.52–2.29) compared to those without overweight or obesity (Fig. 2). All the studies reported a trend of higher risk for type 1 diabetes among those with overweight or obesity, which was significant in four studies (Table 2). Heterogeneity between the studies was low to moderate (I^2^ = 36%, p = 0.16). Funnel plots of study precision vs. the magnitude of association are shown in Additional file 1: Figure S1. Visual inspection shows little evidence of asymmetry, indicating the absence of substantial publication bias. Five studies analyzed separately the obesity and overweight categories; the pooled RR for incident type 1 diabetes in those with obesity was 2.17 (1.75–2.69) (I^2^ = 0%). Three studies distinguished the overweight from obesity category; the pooled RR for incident type 1 diabetes in those with overweight was 1.35 (0.93–1.97) (I^2^ = 45%) (Additional file 1: Figure S2). Sequential exclusion of studies did not materially change the results (Additional file 1: Figure S3). The results persisted regardless of selection between duplicate studies [1.85 (1.45–2.35)] (Additional file 1: Figure S4), and whether fixed or random effects models were applied [1.83 (1.59–2.11)] (Additional file 1: Figure S5).

Three of the seven studies with categorical BMI data required multiple autoantibody data as part of the type 1 diabetes definition. These included 577,744 men and women, and 752 cases of type 1 diabetes (0.13%). The pooled RR for incident type 1 diabetes among those with obesity or overweight/obesity was 2.55 (1.11–5.86) (I^2^ = 80%). Two of these three studies examined obesity separately from the overweight category; the pooled RR for incident type 1 diabetes in those with obesity was 3.57 (2.02–6.32) (I^2^ = 0%) (Fig. 2).

In a subgroup analysis, the risk for incident type 1 diabetes among people with obesity or overweight/obesity was similar in those with long (> 12 years) [n = 2; 1.93 (1.48–2.52), I^2^ = 0%] or short follow-up duration [n = 5; 1.84 (1.36, 2.48), I^2^ = 52%] (Additional file 1: Figure S6). The pooled risk ratio was higher for those younger than 11 years [n = 2; 2.24 (1.00, 5.02), I^2^ = 0%], compared with older age at enrolment [n = 5; 1.85 (1.47, 2.34), I^2^ = 52%], but differences were not statistically significant (Additional file 1: Figure S7). Five studies included populations with an average risk for developing type 1 diabetes (as opposed to high-risk populations). The pooled RR for incident type 1 diabetes was 2.07 (1.74, 2.45) (I^2^ = 0%), compared with 1.38 (1.07–1.79) (I^2^ = 0%) for those with high baseline risk.

### Continuous BMI data

**Fig. 3:**
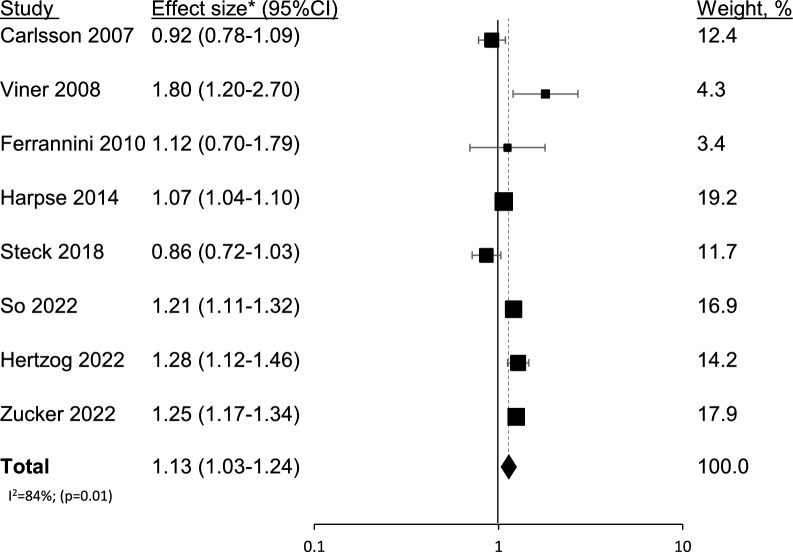
Confounder-adjusted size effect (95% CI) for incident type 1 diabetes for 1 BMI unit increment is shown for individual studies and for pooled risk ratio results from the meta-analysis. The sizes of the boxes correspond to the weight of the study in the meta-analysis. The diamond depicts the point estimate (95% CI). The vertical line is centered at the null. This analysis included 8 studies: 3 that used continuous BMI data only, and 5 that used both continuous and categorical data. Instead of Tosur et al. [20], this analysis included So et al. [17], the most recent publication of the TrialNet PTP cohort. *All the studies reported hazard ratios, except Ferrannini [16], who reported risk ratios. The latter provided only a risk ratio stratified by sex, the measure used in this analysis was that reported in men, which was smaller than that reported in women. The calculation using the RR reported in women did not materially change the results [1.15 (1.04–1.28)]

Eight studies used continuous BMI data: three used only continuous data and were not included in the previous analysis, and five used both continuous and categorical data. The eight studies provided information on 1,620,390 men and women, and 2,108 cases of incident type 1 diabetes (0.13%). The pooled estimate yielded a 13% increased risk for type 1 diabetes per BMI unit increment, with high heterogeneity between the studies (1.13 (1.03–1.24), I^2^ = 84%) (Fig. 3).

## Discussion

In this systematic review and meta-analysis of high-quality observational cohort studies that included nearly 1.7 million individuals, we report a pooled RR of 1.87 (1.52–2.29) for incident type 1 diabetes in children and adults with obesity or overweight/obesity. The point estimates showed a graded risk: RR 1.35 [0.93–1.97] for those with overweight and 2.17 [1.75–2.69] for those with obesity. The point estimates remained stable in sensitivity analyses that addressed potential variability in medical aspects of the association, duration of the follow-up, and misclassification related to exposure or the outcome definition. Specifically, we report a pooled RR of 2.55 (1.11–5.86) for cohort studies in which type 1 diabetes was verified with autoantibody data.

This work has several limitations. First, we used adjusted effect estimates, although the model was adjusted differently across studies. Yet, the effect estimates were comparable among studies that reported results from both adjusted and unadjusted models [15, 23]. Second, the reference group in the risk calculation varied between the studies, as some used normal BMI as a reference while others used a dummy variable; this variability could affect the pooled estimate. However, we observed a graded relation between BMI and incident type 1 diabetes, as the risk increased with higher BMI categories. In addition, the association persisted when BMI was modeled as a continuous variable. Third, the lack of systematic autoantibody data in all the included studies is a major limitation, as misclassification of type 2 diabetes as type 1 diabetes can erroneously accentuate obesity-dependency of the association. This concern is mitigated by a subgroup analysis that showed consistency of the association in studies with autoantibody-proven cases of type 1 diabetes. Fourth, although we excluded studies in which diagnosed diabetes mellitus was present at the beginning of the follow-up, the presence of undiagnosed type 1 diabetes may have been possible. We addressed this issue by including studies with at least 12 months of follow-up and conducting a subgroup analysis by the duration of follow-up; nonetheless this remains a limitation. Fifth, the populations included in the pooled analysis varied in their baseline risk for incident type 1 diabetes and included average-risk and high-risk populations (based on multiple autoantibody positivity, family history of type 1 diabetes, or HLA defied risk). Although we conducted a subgroup analysis based on the type of baseline population, our inability to control for the extent of that risk, both by heritable factors and by comorbidities, is a limitation. Of note, one study in the meta-analysis reported a stratified analysis that considered autoimmune comorbidities at baseline and reported no substantial change to the original point estimates [15]. Sixth, the use of BMI instead of other measures of adiposity is a limitation. Notably, BMI is a valid measure of childhood and adolescent obesity [8], and is the measure preferred by the ADA for weight surveillance of pre-diabetes and diabetes [9]. Finally, as most studies did not stratify estimates by sex, we were unable to explore sex-dependent differences. Notably, two studies in the systematic review included a sex-stratified analysis. One showed a similar risk [15], while the other showed only a slightly elevated risk among females with overweight and obesity [16]. This is consistent with reports that boys and girls are equally affected by type 1 diabetes [2, 26].

In recent decades, several observations have supported the importance of environmental and behavioral factors in the pathogenesis of type 1 diabetes. Among these are the parallel rise in type 1 diabetes [27] and childhood obesity. Ecological [4, 5], case-control [28, 29], and cohort studies [15, 19, 22, 30, 31] have linked obesity with type 1 diabetes. A previous systematic review and meta-analysis that examined the association between childhood obesity and type 1 diabetes reported a pooled OR of 2.03 (95% CI 1.46–2.80). Yet, that meta-analysis included case-control studies, and therefore could not assess relative risk. It also included high birth weight data as the exposure, and therefore narrowed the contribution of environmental aspects related to childhood obesity. These shortcomings are addressed in the design of the current study.

A notable finding in the current study is the graded increase in pooled estimates, as the BMI categories increased. Together with the temporality of events, this strengthens the plausibility of a causal relation, and corroborates a recent Mendelian randomization study [32]. In that study the investigators reported an OR of 1.32 (95%CI 1.06–1.64) per standard deviation score in BMI, using single nucleotide polymorphisms associated with childhood adiposity in children aged 2–10 years. This is especially noteworthy as type 1 diabetes is still considered by many experts a non-obesity related morbidity [33].

Several biologic mechanisms have been proposed for the relation between BMI and type 1 diabetes, some are mediated by insulin resistance, while others involve the proinflammatory state promoted by and the direct toxic effect of the adipose tissue. The “accelerator hypothesis” or the wider “ꞵ-cell stress hypothesis” [34] suggests that biological factors such as overweight and obesity trigger increased insulin demand leading to endoplasmic reticulum stress. This alters insulin synthesis and may lead to ꞵ-cell apoptosis [35]. According to this hypothesis, the increased insulin demand also renders ꞵ-cells susceptible to immune mediated injury. High rate of insulin production may result in the formation of neo-autoantigens created via post-translational modification of islet proteins, which attract immune cells and drive an autoimmune disease process [36]. Obesity has been linked with other autoimmune diseases [24, 37]. A possible explanation is that elevated levels of pro-inflammatory cytokines and adipokines associated with obesity facilitate chronic sub-clinical inflammatory processes, which in turn diminish “self-tolerance” [38]. Additionally, other factors associated with obesity, might promote an autoimmune process, such as consumption of high fat diet, vitamin D deficiency and alterations in gut microbiota [39]. Moreover, animal studies suggest that adipocyte derived hormones might also mediate ꞵ-cells damage specifically. Leptin, which is upregulated in obesity, has been shown to accelerate destruction of ꞵ-cells [40], while adiponectin, that is downregulated in obesity, was shown to protect ꞵ-cells from apoptosis [41]. Finally, given the abundant of mechanisms, it is possible that all contribute to the association to a degree, while the interplay between these differs in various setting and/or populations.

We report a significant pooled estimate, both in populations at average risk for incident type 1 diabetes and with a genetically high-risk (and high incidence). This underlines the importance of maintaining a healthy weight, not only for genetically predisposed individuals. While maintaining a healthy weight is a universally valid recommendation, our findings suggest that targeted public health interventions aimed at reducing childhood obesity might specifically mitigate the growing burden of type 1 diabetes. In particular, the significant point estimates reported in the average-risk populations suggest that the degree of obesity may be involved in the pathogenesis of type 1 diabetes. This is in line with observations of increasing type 1 diabetes incidence among genetically low-risk individuals [42], in parallel with the rising prevalence of childhood and adult obesity [43, 44]. However, unmeasured confounders associated with obesity should be considered, as they might drive the relationship seen with type 1 diabetes. In such case, maintaining healthy weight might not be preventive/therapeutic, but nonetheless important.

The importance of BMI lies not only in the primary prevention but also in the nature of the disease progression [45]. Immunological-related treatments, such as Teplizumab, are available to delay the clinical onset of type 1 diabetes in people with antibodies but without dysglycemia [46]. Although the published studies are small, the level of response was heterogenous, and BMI was identified as one of its determinants [47]. Furthermore, high BMI in people with type 1 diabetes was shown to be accompanied by a more aggressive clinical course that included incident morbidity related to cardiovascular disease and cancer [45].

## Conclusion

In conclusion, in this systematic review and meta-analysis of high-quality observational cohort studies, high BMI was associated with an increased risk of incident type 1 diabetes. These results provide real world evidence for the deleterious contribution of obesity to the development of type 1 diabetes.

### Supplementary Information

**Additional file 1: Figure S1**. Funnel plots for the random-effects model (left) and the fixed-effects model (right). **Figure S2.** Pooled risk ratios (95% CI) obesity and overweight. **Figure S3.** Pooled risk ratio (95% CI) with serial exclusion of each study in turn. **Figure S4.** Pooled risk ratio (95% CI) with alternating duplicates. **Figure S5.** Pooled risk ratio (95% CI) with fixed-effect models. **Figure S6.** Pooled risk ratios by follow up duration (< 12 years, and ≥ 12 years). **Figure S7.** Pooled risk ratios by age at enrollment (< 11 years, and ≥ 11 years). **Figure **S8. Pooled risk ratios by the baseline risk of the population for type 1 diabetes, average vs. high risk. **Tables S1.** Search structures, Medical Subject Headings (MeSH), and keywords used for Ovid MEDLINE and EMBASE databases. **Table S2.** A list of the excluded studies and the reasons for exclusion.

## Data Availability

The datasets used and/or analyzed during the current study are available from the corresponding author on reasonable request.
