# Supplementary material for: High BMI and the risk for incident type 1 Diabetes Mellitus: a systematic review and meta-analysis of aggregated cohort studies
Source: Cardiovasc Diabetol. 2023 Nov 2;22:300. doi: 10.1186/s12933-023-02007-y (PMC10623818; doi:10.1186/s12933-023-02007-y)
Supplement: Supplementary file 1 — Additional file 1: Figure S1. Funnel plots for the random-effects model (left) and the fixed-effects model (right). Figure S2. Pooled risk ratios (95% CI) obesity and overweight. Figure S3. Pooled risk ratio (95% CI) with serial exclusion of each study in turn. Figure S4. Pooled risk ratio (95% CI) with alternating duplicates. Figure S5. Pooled risk ratio (95% CI) with fixed-effect models. Figure S6. Pooled risk ratios by follow up duration (< 12 years, and ≥ 12 years). Figure S7. Pooled risk ratios by age at enrollment (< 11 years, and ≥ 11 years). Figure S8. Pooled risk ratios by the baseline risk of the population for type 1 diabetes, average vs. high risk. Tables S1. Search structures, Medical Subject Headings (MeSH), and keywords used for Ovid MEDLINE and EMBASE databases. Table S2. A list of the excluded studies and the reasons for exclusion. [file 12933_2023_2007_MOESM1_ESM.docx]

**High BMI and the Risk for Incident Type 1 Diabetes Mellitus: a Systematic Review and Meta-analysis of Aggregated Cohort Studies**

**Supplementary Materials**

[Table S1: Literature search strategy](#_Tables_S1:_)………………………………………………………….2

[Table S2: A list of the excluded studies and the reasons for exclusion](#_Table_S2:_List) ……….…………….4

[Figure S1: Funnel plots](#_Figure_S1:_Funnel)……………………………………………………………………....6

[Figure S2: Pooled RR obesity and overweight](#_Figure_S2:_Pooled)……………………………………………....6

[Figure S3: Pooled RR with serial exclusion of each included study](#_Figure_S3:_Pooled)…………………………7

[Figure S4: Pooled RR with alternating between duplicates](#_Figure_S4:_Pooled) ..………………………………..7

[Figure S5: Pooled RR with fixed-effect model](#_Figure_S5:_Pooled) …...…………………………………………8

[Figure S6: Pooled RR by follow up duration](#_Figure_S6:_Pooled) ………...……………………………………...8

[Figure S7: Pooled RR Pooled RR by age at enrollment](#_Figure_S7:_Pooled) ……...……………………………...9

[Figure S8: Pooled RR by the baseline risk of the population for T1DM (average vs. high)](#_Figure_S8:_Pooled) ...9

**Search strategy:**

The MeSH terms "Obesity", "Obese", "Overweight", "Weight", and "Body Mass Index"/"BMI" were combined with the operator ‘OR’. The MeSH terms "Type 1", "Type I", "early onset", and "juvenile" diabetes were combined using the operator "OR". This term incorporates all the terms that have been used to describe this disease, including insulin-dependent diabetes, juvenile diabetes, and autoimmune diabetes. The MeSH term "risk" was entered. The MeSH terms "cohort", "prospective", "retrospective", "longitudinal", "follow" and "followed" were combined using the operator "OR". These four elements were combined using the operator ‘AND’.

# Tables S1: Search structures, Medical Subject Headings (MeSH), and keywords used for Ovid MEDLINE and EMBASE databases

|  | Ovid MEDLINE(R) and Epub Ahead of Print, In-Process, In-Data-Review & Other Non-Indexed Citations and Daily |
| --- | --- |
| 1 | (('type 1' or 'type i' or 'early onset' or juvenile) adj2 diabetes).mp. [mp=title, abstract, original title, name of substance word, subject heading word, floating sub-heading word, keyword heading word, organism supplementary concept word, protocol supplementary concept word, rare disease supplementary concept word, unique identifier, synonyms] |
| 2 | (obesity or obese or overweight or weight or 'body mass' or bmi or 'body size').mp. |
| 3 | risk.mp. |
| 4 | (cohort* or prospective* or retrospective* or longitudinal or follow or followed).mp. |
| 5 | 1 and 2 and 3 and 4 |
|  | EMBASE |
| #1 | 'insulin dependent diabetes mellitus'/de |
| #2 | (('type 1' OR 'type i' OR 'early onset' OR juvenile) NEAR/2 diabetes):ti,ab,kw |
| #3 | #1 OR #2 |
| #4 | obesity OR obese OR overweight OR weight OR 'body mass' OR bmi OR 'body size' |
| #5 | risk |
| #6 | cohort* OR prospective* OR retrospective* OR longitudinal OR follow OR followed |
| #7 | #3 AND #4 AND #5 AND #6 |

## **Table S2: A list of the excluded studies and the reasons for exclusion**

| Index | Author | Reason (number of excluded studies) |
| --- | --- | --- |
| 1 | Johansson 1994 | Irrelevant study design (non-cohort) |
| 2 | Hypponen 2000 | (n=13) |
| 3 | Bruining 2000 |  |
| 4 | Bruno 2005 |  |
| 5 | Knerr 2005 |  |
| 6 | Knip 2008 |  |
| 7 | Waldhoer 2008 |  |
| 8 | Winkler 2012 |  |
| 9 | Antvorskov 2018 |  |
| 10 | Jacobsen 2019 |  |
| 11 | Lawrence 2021 |  |
| 12 | Morales 2021 |  |
| 13 | Räisänen 2022 |  |
| 14 | Hemminki 2012 | Irrelevant exposure |
| 15 | Larsson 2016 | (n=5) |
| 16 | Li 2020 |  |
| 17 | Liu 2020 |  |
| 18 | Li 2022 |  |
| 19 | Chien 2008 | Irrelevant outcome |
| 20 | AlMamun 2009 | (n=5) |
| 21 | Olsson 2009 |  |
| 22 | Lee 2012 |  |
| 23 | Beyerlein 2014 |  |
| 24 | Ferrara 2017 | Reported on BMI change or velocity |
| 25 | Ferrara 2017 | (n=3) |
| 26 | Lamb 2009 |  |
| 27 | Ferrannini 2010 | Abstracts of an included full text (duplicate) |
| 28 | Ferrara 2016 | (n=2) |
| 29 | Abbasi 2017 | Insufficient median follow-up time |
| 30 | Pflueger 2012 | Exposure data under the age of 2 years (n=1) |

## **
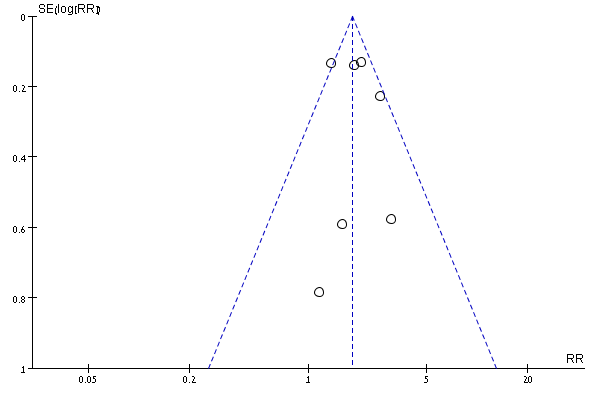
Figure S1: Funnel plots for the random-effects model (left) and the fixed-effects model (right)**

**
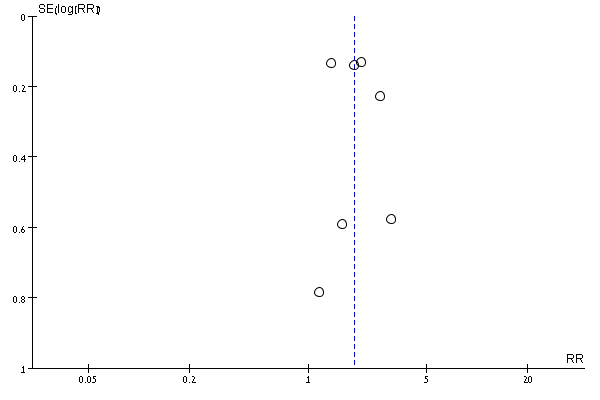
**

The lack of substantial asymmetry in the distribution of association estimates (black dots) around the pooled estimate (vertical dashed blue line) suggests the absence of substantial publication bias. The dashed triangle borders (right) depict the pseudo-95% CI around the pooled estimate. RR – risk ratio, SE, standard error.

## **Figure S2: Pooled risk ratios (95% CI) obesity and overweight**

(a)

**
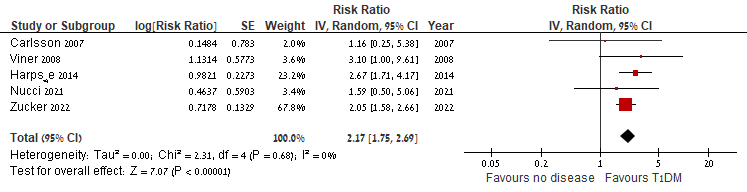
**
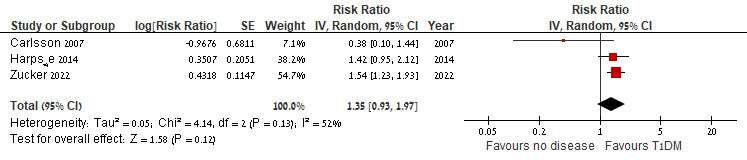


(b)

Confounder-adjusted pooled effect estimates (95% CI) for incident type 1 diabetes among individuals with obesity (a) or overweight (b) compared to those without, are shown for the individual studies and for the pooled risk ratios result from the meta-analysis. The sizes of the boxes correspond to the weights of the studies in the meta-analysis. The diamonds depict the pooled point estimates (95% CI). The vertical lines are centered at the null. Obesity was defined as follows: Carlsson (2007) and Harpsøe (2014) – BMI ≥30 kg/m^2^, Viner (2008) and Nucci (2021) – according to the International Obesity Task Force cut-off points (corresponding to BMI≥30 kg/m^2^ in adults), Zucker (2022) – BMI≥95th percentile. Overweight was defined as follows: Carlsson (2007) and Harpsøe (2014) – BMI≥25 kg/m^2^, Zucker (2022) – 85th≤BMI≤94th percentiles.

## **Figure S3: Pooled risk ratio (95% CI) with serial exclusion of each study in turn**


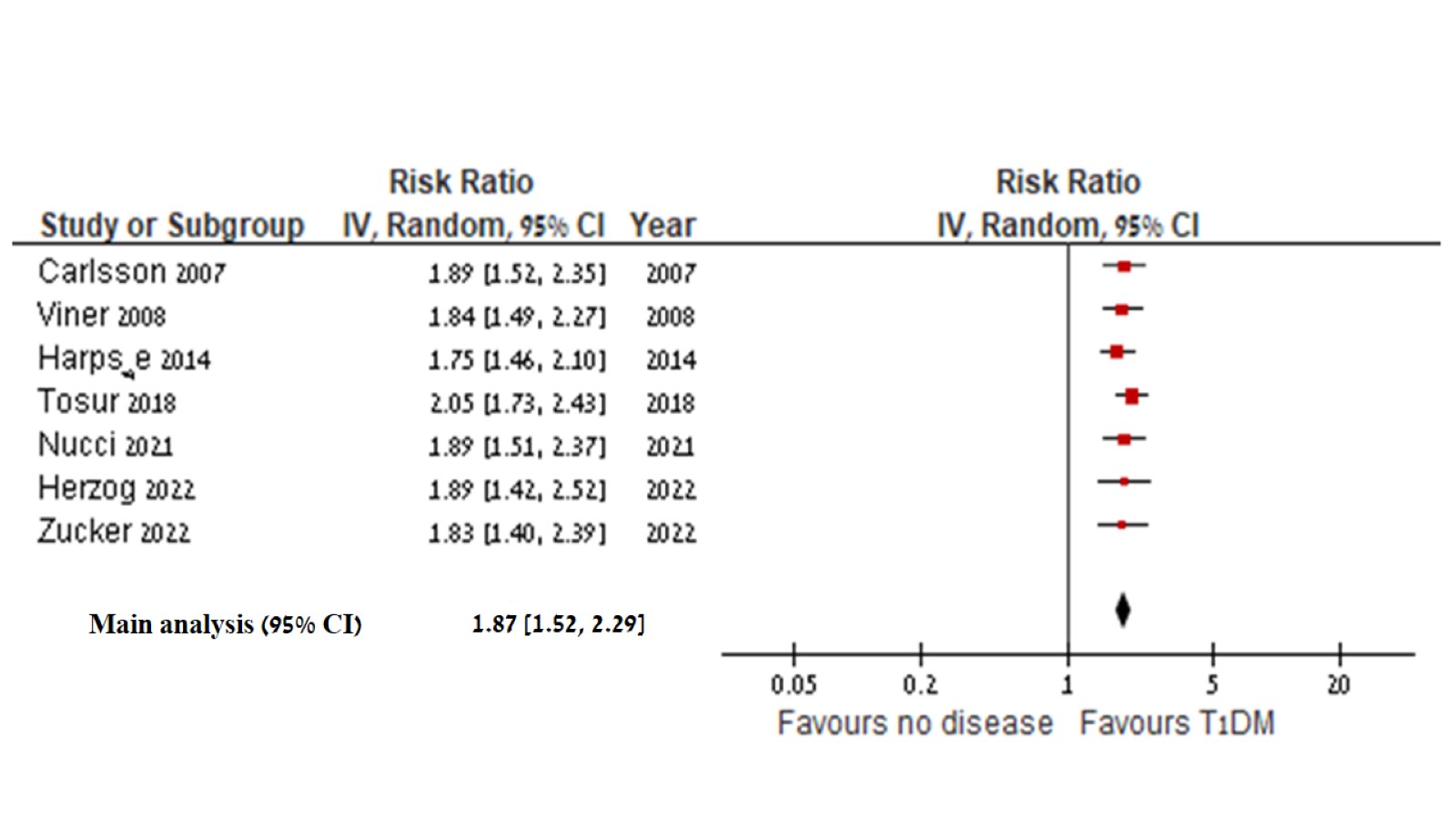
Pooled effect estimates (95% CI) for incident type 1 diabetes among individuals with obesity or overweight/obesity compared to those without are shown for the sequential exclusion of included studies. The pooled risk ratios result of the meta-analysis, including all seven studies, is shown at the bottom. The sizes of the boxes correspond to the weights of the studies in the meta-analysis. The diamond depicts the pooled point estimate (95% CI). The vertical line is centered at the null.

## **Figure S4: Pooled risk ratio (95% CI) with alternating duplicates**


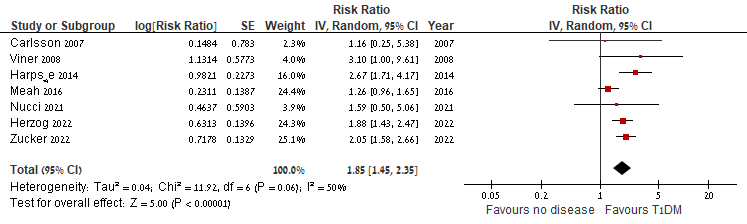


Confounder-adjusted pooled effect estimates (95% CI) for incident type 1 diabetes among individuals with obesity or overweight/obesity compared to those without, are shown for the individual studies and for the pooled risk ratio result from the meta-analysis. In this analysis, Meah 2016 was included instead of Tosur 2018 (1.87 [1.52-2.29]). The sizes of the boxes correspond to the weights of the studies in the meta-analysis. The diamond depicts the pooled point estimate (95% CI). The vertical line is centered at the null.

## **Figure S5: Pooled risk ratio (95% CI) with fixed-effect models**


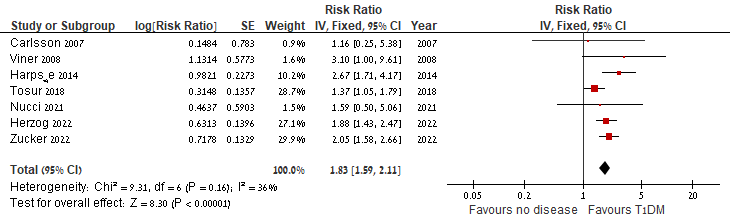


Confounder-adjusted pooled effect estimates (95% CI) for incident type 1 diabetes among individuals with obesity or overweight/obesity compared to those without, are shown for the individual studies and for the pooled risk ratio result from the meta-analysis using fixed-effect models. The sizes of the boxes correspond to the weights of the studies in the meta-analysis. The diamond depicts the point estimate (95% CI). The vertical line is centered at the null.

## **
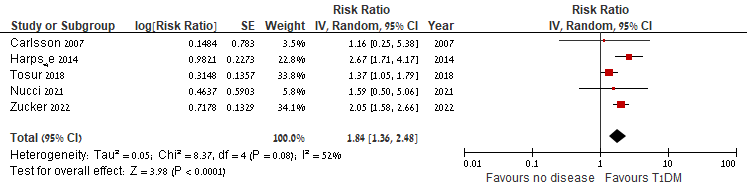
Figure S6: Pooled risk ratios by follow up duration (<12 years, and ≥12 years)**

(a)


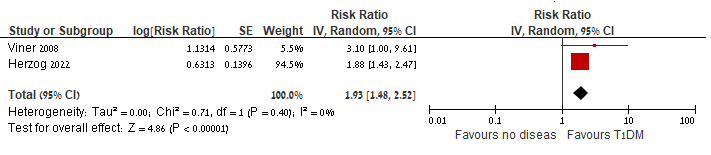


(b)

Confounder-adjusted pooled effect estimates (95%CI) for incident type 1 diabetes among individuals with obesity or overweight/obesity compared to those without, are shown for the individual studies and for the pooled risk ratio results from the meta-analysis. The studies were stratified by follow-up duration up to 12 years (a), and for 12 years and longer (b). The sizes of the boxes correspond to the weights of the studies in the meta-analysis. The diamond depicts the pooled point estimate (95% CI). The vertical line is centered at the null.

## **Figure S7: Pooled risk ratios by age at enrollment (<11 years, and ≥11 years)**

(a)


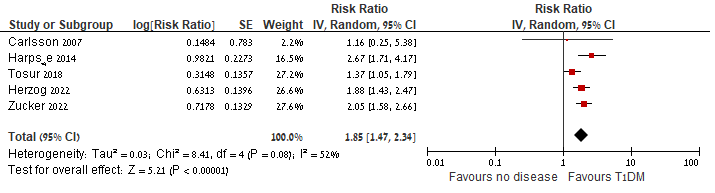
**
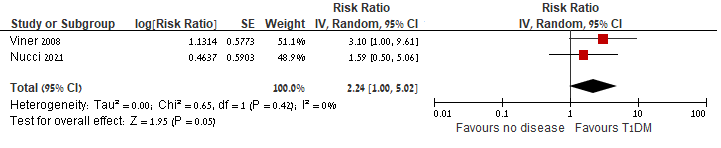
**

(b)

Confounder-adjusted pooled effect estimates (95% CI) for incident type 1 diabetes among individuals with obesity or overweight/obesity compared to those without, are shown for the individual studies and for the pooled risk ratio results from the meta-analysis. The studies were stratified by age at enrollment < 11 years (a) and *>* 11 years (b). The sizes of the boxes correspond to the weights of the studies in the meta-analysis. The diamond depicts the pooled point estimate (95% CI). The vertical line is centered at the null.

## **Figure S**8**: Pooled risk ratios by the baseline risk of the population for type 1 diabetes, average vs. high risk**

(a)


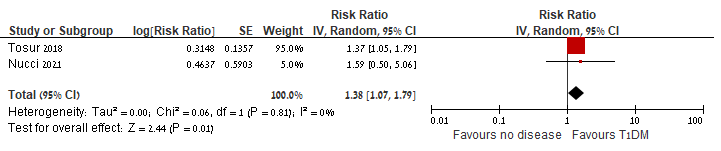

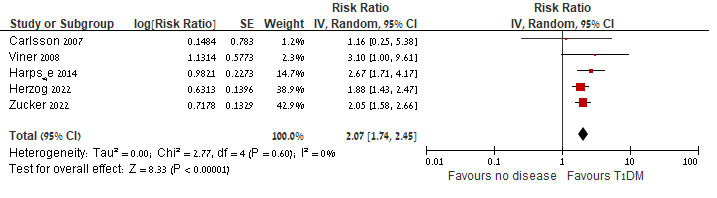


(b)

Confounder-adjusted pooled effect estimates (95% CI) for incident type 1 diabetes among individuals with obesity or overweight/obesity compared to those without, are shown for the individual studies and for the pooled risk ratios result from the meta-analysis. The studies were stratified by the baseline risk of the population for type 1 diabetes: average-risk (a) and high-risk (b). The sizes of the boxes correspond to the weights of the studies in the meta-analysis. The diamond depicts the pooled point estimate (95% CI). The vertical line is centered at the null.
